# Responses of Morphology, Gas Exchange, Photochemical Activity of Photosystem II, and Antioxidant Balance in *Cyclocarya paliurus* to Light Spectra

**DOI:** 10.3389/fpls.2018.01704

**Published:** 2018-11-21

**Authors:** Yang Liu, Tongli Wang, Shengzuo Fang, Mingming Zhou, Jian Qin

**Affiliations:** ^1^College of Forestry, Nanjing Forestry University, Nanjing, China; ^2^Department of Forest and Conservation Sciences, University of British Columbia, Vancouver, BC, Canada; ^3^Co-Innovation Center for Sustainable Forestry in Southern China, Nanjing Forestry University, Nanjing, China

**Keywords:** *Cyclocarya paliurus*, light quality, morphology, pigments content, PSII activity, antioxidant balance

## Abstract

Light quality is a critical factor regulating photosynthetic capacity which directly affects the final yield of plants. *Cyclocarya paliurus* is a multiple function tree species and its leaves are widely used as tea production and ingredient in functional foods in China. However, the effects of varying light quality on photosynthetic process and the photoprotective mechanisms remains unexplored in-depth. In this study, the biomass accumulation, morphology changes, photosynthetic capacity, stomata ultrastructure, pigments content, PSII activity, reactive oxygen species production, antioxidant enzymes, and phenolic content of *C. paliurus* plants under different light-emitting diodes (LED) light treatments were investigated to test a hypothesis that the difference in photosynthetic efficiency of *C. paliurus* plants under differential light quality is related to the degree of photoinhibition and the activation of photoprotection. We found that *C. paliurus* plants performed better under the treatments of WL (white light, 445 and 560 nm) and BL (blue light, 456 nm) than the treatment of GL (green light, 514 nm) and RL (red light, 653 nm). The better performances were characterized by higher values of photosynthetic capacity, total biomass, pigments content, specific leaf mass per area, seeding height increment, leaf thickness and palisade length. In contrast, plants under the treatments of GL and RL suffered significant photoinhibition but effectively developed photoprotective mechanisms. Results of this study provide not only some insights of the response mechanisms of plant photosynthesis to light quality but also a scientific basis for improving the cultivation of *C. paliurus* plantations.

## Introduction

*Cyclocarya paliurus* (Batal) Iljinskaja is a multiple function tree species that belongs to the Juglandaceae family and is mainly distributed in the sub-tropical highlands of China (Fang et al., 2006). Leaves of this species have been widely used as tea production and ingredient of food industry for a long time in China (Fang et al., 2011; Xie et al., 2015). Diverse biological activities, including antioxidant, antidiabetic and antimicrobial activities, have been found in the extracts of the leaves, which are attributed to the abundant phytochemicals such as flavonoids, triterpenoids, polyphenolics, and polysaccharides (Kurihara et al., 2003; Xie et al., 2015; Wu et al., 2017; Liu et al., 2018a,b). Because of these beneficial effects on the human health, an increased demand for the production of *C. paliurus* leaves is now required for medical and commercial uses. Thus, large-scale leaf-harvesting plantations of this species have been established in recent years, and cultivation technologies including optimizing soil and light environment have been carried out (Deng B. et al., 2012; Liu et al., 2016).

Light is considered the most direct environmental factor affecting plant growth and development (Ma et al., 2015; Yamori, 2016). Specifically, changes in light wavelengths due to the different properties of light-harvesting pigments can directly affect plant morphological, anatomical, biochemical, and physiological parameters (Haliapas et al., 2008; Fan et al., 2013). Red light has been shown to control the development of photosynthetic apparatus and up-regulate the integrity of starch in some species (Sæbø et al., 1995; Shimizu et al., 2011). Blue light is reported to control the integrity of chloroplast proteins, which are important for chloroplast development and plant photosynthesis (Hogewoning et al., 2010). Meanwhile, distinct effects of green light on leaf hyponasty, stem elongation, apical dominance, leaf expansion, and photosynthesis have been reported (Schmitt and Wulff, 1993; Wang and Folta, 2013). Previous studies also reported that responses of plants to differential light quality are species specific (Hogewoning et al., 2010; Nanya et al., 2012; Yu et al., 2017). For example, Ouyang et al. (2003) reported that blue light resulted in significantly higher biomass accumulation of *Cistanche deserticola* than red light. However, Chen et al. (2014) showed that fresh weight and dry weight of lettuce were lowest under blue light when compared with other light treatments. Yu et al. (2017) also reported that red light improved *Camptotheca acuminata* growth by reducing the accumulation of reactive oxygen species (ROS) and activating photosynthetic processes. Additionally, Johkan et al. (2012) reported that short-wavelength green LED light with high intensity was effective to active leaf photosynthetic rate and plant growth of *Lactuca sativa*.

Plants have developed several useful photoprotective mechanisms to cope with the diverse light stresses such as excess UV light and other non-beneficial light wavelengths (Yamori, 2016). The first photoprotective mechanism is known as avoiding exposure to light through some simple methods like leaf movement, changing of leaf area (LA), and altering chloroplast positions (Corlett et al., 1994; Suetsugu and Wada, 2007). Photoprotective mechanisms involved in the photosynthetic electron transport have also been proposed, including (1) thermal energy dissipation to protect PSII [also called non-photochemical quenching (NPQ)], (2) plastoquinol terminal oxidase (PTOX) as an alternative electron transport around PSI, and (3) ROS scavenging mechanisms through non-enzymatic antioxidants (ascorbate, carotenoids, and tocopherol) and enzymatic antioxidants [including superoxide dismutase (SOD), peroxidase (POD), and catalase (CAT)] (Baena-Gonzalez and Aro, 2002; Holt et al., 2004; Takahashi and Badger, 2011). Among these protective systems, NPQ mechanism and ROS scavenging abilities are often measured to identify the adaptation level of plants under light stress, as they can effectively reduce the damage caused by excessive light energy (Yamori, 2016; Yu et al., 2017). It should be noted that some plants also produce other macromolecular compounds like phenolics or flavonoids to scavenge ROS caused by light stress, especially when UV or short-wavelength green and blue lights increased (Cruces et al., 2017; Liu et al., 2018c).

Nowadays, some spectrum LEDs or films have been used in indoor plant pre-cultivation (or in greenhouse) at seedling developmental stage to promote the growth traits and the accumulation of bioactive compounds in economic or medicinal trees, such as *Quercus ithaburensis* and *Anoectochilus roxburghii* (Smirnakou et al., 2017; Ye et al., 2017). Previous studies have reported the effects of differential light quality and light intensity on the development of *C. paliurus*, such as biomass and polysaccharide accumulation (Yang et al., 2017). Moreover, it was demonstrated that blue light promoted the production of functional flavonoids (kaempferol, isoquercitrin, and quercetin) and related gene expression in *C. paliurus* seedlings (Liu et al., 2018c). However, little information is known about how varying light quality affects the photosynthetic process and the photoprotective mechanisms in *C. paliurus* seedlings. We hypothesized that the difference in photosynthetic efficiency of plants under differential light quality was related to the degree of photoinhibition and the activation of photoprotection, which occur at both morphology and photochemical levels including anatomical changes, the regulation of photochemistry activity and photosynthetic pigments, as well as antioxidant balance including enzymes systems and non-enzymes systems. Thus, the objective of this study was to investigate the response mechanisms of growth and photosynthesis of *C. paliurus* to varying light qualities (white, blue, green and red LED lights) through measuring biomass accumulation, morphology changes, photosynthetic capacity, stomata ultrastructure, pigments content, chlorophyll fluorescence parameters, ROS production, antioxidant enzymes, and phenolic content. Findings from this study will give insights into the response mechanisms of plant photosynthesis to differential light quality and provide scientific basis for cultivation of this plant species with supplementary artificial light, especially at seedling developmental stage.

## Materials and Methods

### Plant Materials and Light Treatments

The experiment under different light treatments was carried out in a controlled climate chamber at Nanjing Forestry University (31° 59′ N, 119° 18′ E) during the growing season in 2016. Seeds of *C. paliurus* were obtained from Anji (Zhejiang province, China; 27° 46′ N, 119° 17′ E) and germinated according to the method of Fang et al. (2006) in Baima experimental base of Nanjing Forestry University (31° 23′ N, 118° 51′ E). Three months later, *C. paliurus* seedlings were transplanted into non-woven containers (10.0 cm height, 8.0 cm diameter). The non-woven containers were filled with a substrate mixture of soil: perlite: peat: fowl manure (2: 4: 2: 2, v/v/v/v. pH 6.44). The substrate was imported with an organic matter content of 73.2 g kg^-1^, total P content of 2.20 g kg^-1^, total N content of 72.4 g kg^-1^, and total K content of 9.5 g kg^-1^. Seedlings were watered once a day.

Eight weeks later, 1-year-old homogenous and healthy *C. paliurus* seedlings (diameter 3.0 mm, height 36.5 cm) were selected and moved to the climate chamber at Nanjing Forestry University. Four LED light (Guangdong PHILIPS Lamp Co., China) treatments were used including WL (white LED, maximum 445 and 560 nm, as control), BL (blue LED, maximum 456 nm), GL (green LED, maximum 514 nm), and RL (red LED, maximum 653 nm). The spectral characteristics of the LEDs used were measured using a Ventana 785 spectrometer (Ocean Optics, United States) and showed in the Supplementary Figure S1. Plants of all treatments were kept watered every 2 days with a 12-h photoperiod [25 ± 2°C, 60% relative humidity (RH) at daytime and 21 ± 2°C, 70% RH at night] and a light flux intensity of 800 ± 50 μmol m^-2^ s^-1^ (Li-Cor, LI-6400, United States). Each LED light treatment contained 3 replications and 10 plants per replication.

### Plant Growth Measurement

**FIGURE 1:**
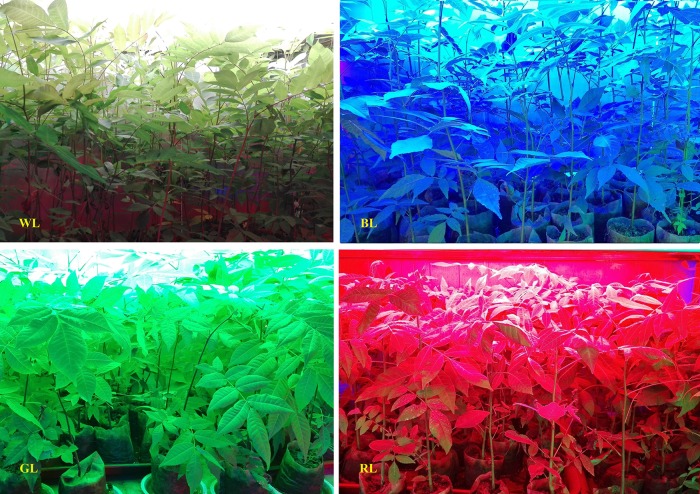
*Cyclocarya paliurus* plants under different LED light treatments (80 days).

After 80 days of treatment, obvious differences of *C. paliurus* seedlings under different LED light treatments were observed (Figure 1). Intact seedlings in each light treatment (3 seedlings) were then harvested and separated into leaves, stems, and roots for biomass and morphology analysis. Biomass was measured by oven-drying roots and shoots at 80°C for 2 days.

### Morphological Analysis

Leaf area (cm^2^) of fully expanded leaves (the third or fourth one from the top of the shoots) was measured before biomass harvest, using an area meter (Li-Cor, Model 3100 area meter, United States) (3 seedlings per treatment). Cross sections of these leaf samples were cut into about 4 mm × 6 mm at the same time, then fixed with FAA, and observed on a Nikon YS100 microscope (Nikon Co., Tokyo, Japan). Leaf thickness, spongy tissues and palisade tissues were measured using a FW4000 software, as the method described previously (Liu et al., 2016). Stem diameter and seedling height were measured at the same time, seedling height increment (SHI) and ground diameter increment (GDI) were calculated by subtracting the final values from the initial values.

### Photosynthetic Parameters

Gas-exchange parameters in fully developed leaves of *C. paliurus* seedlings were measured with a portable photosynthesis system Li-6400R (Li-cor, United States). Measurements were conducted in a 6 cm^2^ leaf chamber under a controlled condition of the air concentration of 21% O_2_, 380 μmol mol^-1^ CO_2_, and 850 μmol m^-2^ s^-1^ photosynthetically active radiation (PAR), 50% relative humidity and the temperature of 25 ± 2°C. Healthy and functional leaves that have a similar size were identified in each treatment and measured (*n* = 3).

### Scanning Electron Microscopy of Stomata

The third or fourth leaf from the top of shoots was immediately harvested from three seedlings in each light treatment after measurement of photosynthetic parameters and immersed in cold sodium cacodylate buffer [0.05 M, with 3% (v/v) glutaraldehyde, pH 7.2]. All samples were then post-fixated, resin embedded in 1% (v/v) osmium acid. Afterward, observations and micrographs were taken with a Philips Model TM-1000 SEM (Hitachi, Japan). The estimation of stomatal size and density in the abaxial surface was carried out according to the methods of Snider et al. (2009). The total stomatal pore area index (SPI) was calculated by multiplying the number of stomata per unit area and the single stomatal opening area.

### Pigment Analysis

To evaluate chlorophyll content, about 0.1 g finely cut leaf sample (from the third or fourth on the top) in each treatment was mixed and extracted with 8 mL 95% alcohol. The extraction of chlorophyll was conducted at 4°C in the dark for 24 h and shaken about three or four times until the leaf samples were blanched. The absorbance of the samples was measured at 649, 665, and 470 nm using a spectrophotometer (Shimadzu UV-2550, Kyoto, Japan). Chlorophyll concentrations were calculated according to the method of Arnon (1949), with three replicates.

### Chlorophyll Fluorescence Parameters

Chlorophyll fluorescence parameters of the leaves under different light qualities were evaluated using a Handy PEA fluorometer (Hansatech Instruments, King’s Lynn, Norfolk, United Kingdom). The JIP-test was carried out as described in a previous study of Pagter et al. (2008), and digitized data of the fast chlorophyll a fluorescence curves were recorded. The OJIP curves were induced by 3,000 μmol m^-2^ s^-1^ pulsing red light (650 nm). The dark adaptation period before the measurement was 15 min (based on our pre-test). The fluorescent parameters of leaf samples, including Fv/Fm (the maximum quantum yield of the primary PSII photochemistry), ABS/RC (the absorbed light energy by the PSII antenna photon flux per active reaction center), DIo/RC (non-photochemical quenching per reaction center of PSII), TRo/RC (total energy used to reduce Q_A_ by the unit reaction center of PSII), photosynthetic performance index (*PI*_ABS_) and non-photochemical quenching per cross section (DIo/CSm) were calculated from the JIP-test curves.

### Determinations of Lipid Peroxidation and H_2_O_2_ Content

Lipid peroxidation of leaves under LED light conditions was assessed as malondialdehyde (MDA) content following the method of Deng Y. et al. (2012) and frozen leaf material (1 g) was used in each replicate (*n* = 3). Similarly, frozen leaf (1 g) was used for the analysis of H_2_O_2_ content in each replicate, following the method of Patterson et al. (1984) (*n* = 3).

### Activities of Antioxidant Enzymes

Crude enzyme extract of each light treatment was obtained from 0.3 g of frozen leaves according to the method of Yu et al. (2017). The supernatant was then collected and used for the measurement of SOD, POD, and CAT enzyme activities.

The SOD activity was analyzed using the method of Giannopotitis and Ries (1977). The POD activity was estimated with the method described by Thomas et al. (1982). Meanwhile, the CAT activity was measured following the method of Díaz-Vivancos et al. (2008). Protein contents in the light treatments were measured at the same time (Pashkovskiy et al., 2018), and the activities of SOD, POD, and CAT in leaves were expressed as U min^-1^ mg^-1^ protein^-1^.

### Quantification of Phenolic Compounds

Phenolic profiles of *C. paliurus* leaves from different light treatments were measured using an HPLC system (Waters, Milford, MA, United States) according to the method in a previous study (Cao et al., 2017) with slight modifications. The separation of *C. paliurus* phenolics was carried out on an X-Bridge C18 column by a stepwise elution with acetonitrile containing 0.01% formic acid (solution A) and water containing 0.01% formic acid (solution B). The gradient elution included 0–13 min, 8% A; 13–28 min, 19% A; and 28–40 min, 21% A. The flow rate was kept at 1.0 ml/min and the injection volume was 10 μl. Meanwhile, the column temperature was kept at 45°C and the wavelength for detection was 205 nm. Contents of individual compounds were quantified from their external standards (Liu et al., 2018d). HPLC chromatograms of a *C. paliurus* sample and the standards can be seen in the Supplementary Figure S2. Total phenolic contents were the sum of all individual phenolics detected.

### Statistical Analysis

An LSD test and analysis of variance (ANOVA, GLM procedure) were performed using SPSS 16.0 (SPSS, Chicago, IL, United States). The data were presented as mean ± standard deviation (SD). Duncan’s multiple range test (*P* = 0.05) was employed to detect differences between light treatments. Differences at *P* < 0.05 were considered significant.

## Results

### Plant Growth

Plants grown under WL had the largest total dry weight and leaf dry weight (Table 1). Total biomass of *C. paliurus* plants was reduced by 4.69, 31.46, and 17.84% (*P* < 0.05) under BL, GL and RL, respectively, compared with WL. However, there was no significant difference of total dry biomass between BL and WL. Compared to WL, Root/Shoot ratios under other light treatments were all significantly increased. BL had the highest root dry weight; however, GL achieved the highest value of Root/Shoot ratio.

**Table 1:** Effects of light quality on biomass accumulation in *C. paliurus* seedlings (Means ± SD).

Treatment	Root biomass (g)	Leaf biomass (g)	Total biomass (g)	Root/Shoot
WL	6.5 ± 0.16b	7.7 ± 0.39a	21.3 ± 0.65a	0.44 ± 0.004b
BL	7.3 ± 0.23a	6.6 ± 0.30b	20.3 ± 0.31a	0.56 ± 0.015a
GL	5.4 ± 0.26c	4.5 ± 0.10d	14.6 ± 0.10c	0.59 ± 0.038a
RL	6.4 ± 0.05b	5.4 ± 0.04c	17.5 ± 0.23b	0.58 ± 0.005a


*Different letters indicate significant differences (*P* < 0.05) between LED light treatments according to ANOVA and LSD test, *n* = 3. LED lamps used: WL, white light; BL, blue light; GL, green light; RL, red light.*

### Morphology

**FIGURE 2:**
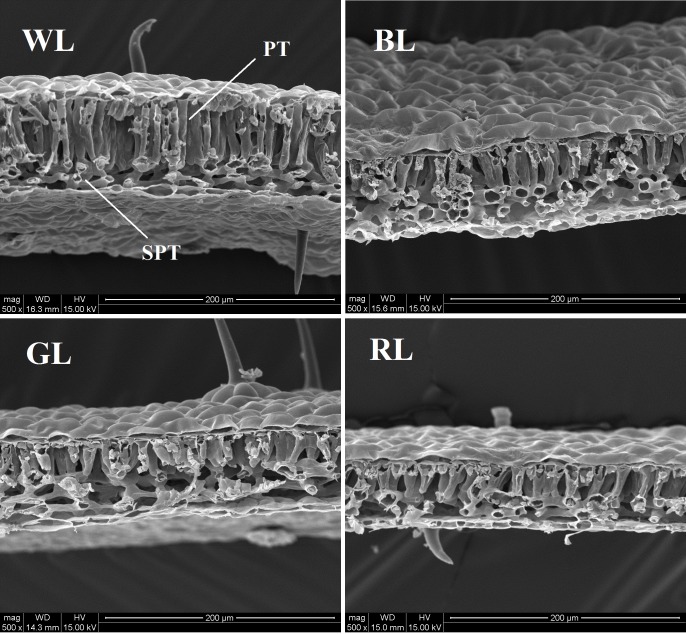
Anatomical structure of *C. paliurus* leaves under different light treatments (PT, palisade parenchyma; SPT, spongy tissue). Scale: 200 μm. LED lamps used: WL, white light; BL, blue light; GL, green light; RL, red light.

Leaf area of plants achieved the highest value under GL, but decreased significantly under BL and RL (*P* < 0.05), compared with WL (Table 2). Specific leaf mass per area (SLM), palisade length, palisade/spongy ratio, and SHI were greatest for seedlings grown under WL, and lowest under GL (Table 2 and Figure 2). Values of leaf thickness under BL, GL and RL were significantly lower than that of WL, with the lowest value obtained from RL (Table 2 and Figure 2). There was no significant difference of GDI among BL, GL and WL, but a significantly higher value was observed under RL.

**Table 2:** Effects of light quality on leaf area (LA), specific leaf mass per area (SLM), leaf thickness, palisade length, palisade/spongy ratio, seedling height increment (SHI), and ground diameter increment (GDI) in *C. paliurus* (Means ± SD).

Treatment	LA (cm^2^)	SLM (g m^-2^)	Leaf thickness (μm)	Palisade length (μm)	Palisade/Spongy	SHI (cm)	GDI (mm)
WL	1720 ± 66.1a	39.6 ± 1.82a	83.6 ± 1.69a	60.3 ± 4.84a	2.8 ± 1.05a	64.7 ± 4.93a	5.2 ± 0.31b
BL	1398 ± 55.0b	32.6 ± 2.21b	74.4 ± 5.57b	41.7 ± 2.80b	1.3 ± 0.25bc	59.0 ± 1.73a	5.3 ± 0.21b
GL	1790 ± 78.3a	18.7 ± 0.98d	67.9 ± 4.78b	28.5 ± 1.98c	0.7 ± 0.14c	41.3 ± 1.53c	5.2 ± 0.15b
RL	1090 ± 53.8c	28.6 ± 1.70c	59.4 ± 0.89c	38.2 ± 1.88b	1.8 ± 0.18ab	50.7 ± 3.79b	6.6 ± 0.55a


*Different letters indicate significant differences (*P* < 0.05) between LED light treatments according to ANOVA and LSD test, *n* = 3. LED lamps used: WL, white light; BL, blue light; GL, green light; RL, red light.*

### Scanning Electron Microscopy of Stomata

**FIGURE 3:**
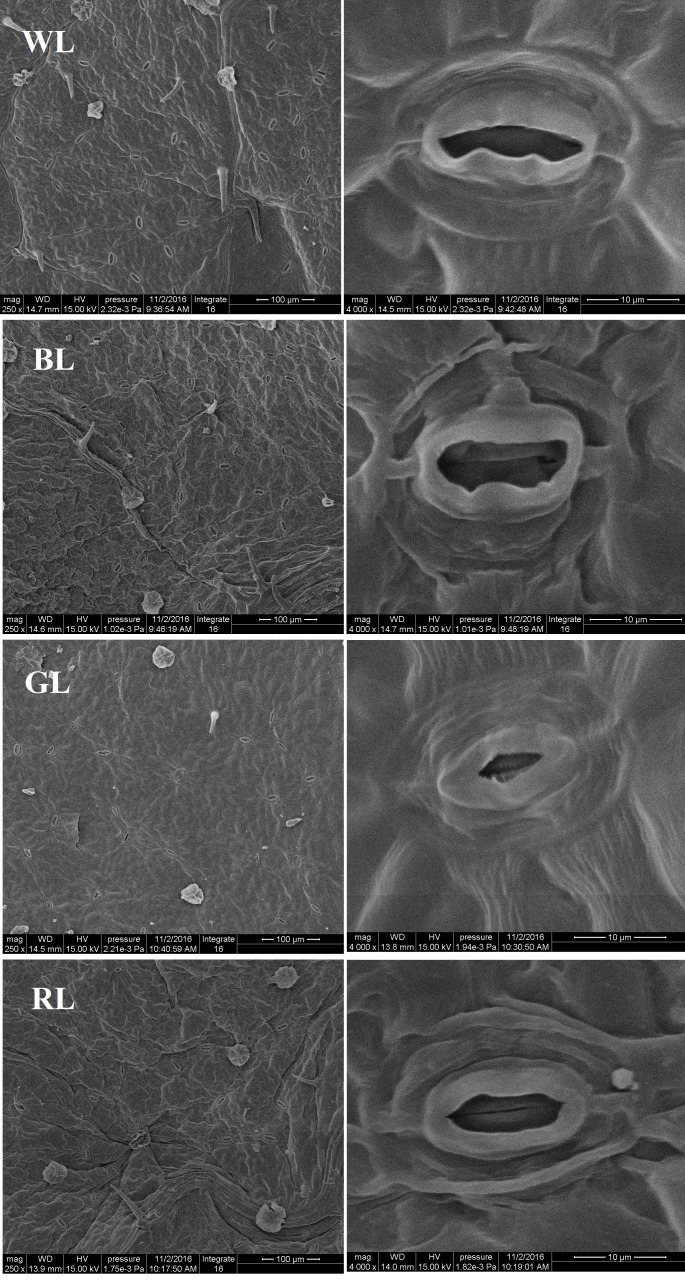
Structure of stomata from *C. paliurus* leaves under different light treatments. Scale: 100 μm **(left)** and 10 μm **(right)**.

Both the stomatal density and size were significantly affected by differential light quality (Figure 3). Values of leaf stomatal length, width and opening were largest under BL and smallest under GL (Table 3). These values under RL and WL were intermediate. Stomatal opening increased by 40.2% under BL, but decreased by 72.9 and 7.6% under GL and RL, respectively, as compared with WL (*P* < 0.05). Meanwhile, the highest stomata number per area was observed in WL-treated leaves and the lowest was found in GL-treated leaves. There was no significant difference in total SPI between WL and BL treatments. However, this parameter was reduced by 85.4% (*P* > 0.05) under GL and by 47.1% under RL, as compared with WL.

**Table 3:** Stomata index of *C. paliurus* leaves under different LED light treatments (Means ± SD).

Treatment	Stomatal length (μm)	Stomatal width (μm)	Single stomatal pore area (μm^2^)	No. of stomata (m m^-2^)	Total stomatal pore area index (SPI) (μm^2^/m^2^)
WL	24.5 ± 0.38b	19.9 ± 0.17b	52.8 ± 1.64b	124.0 ± 9.54a	6559.8 ± 707.74a
BL	29.1 ± 0.36a	24.2 ± 1.07a	74.1 ± 1.52a	90.1 ± 4.86b	6668.9 ± 377.06a
GL	24.7 ± 0.71b	17.2 ± 0.69c	14.3 ± 1.56d	66.7 ± 3.18c	960.1 ± 146.11c
RL	28.9 ± 0.29a	18.1 ± 0.85c	48.8 ± 0.36c	71.0 ± 6.62c	3468.8 ± 346.75b


*Different letters indicate significant differences (*P* < 0.05) between LED light treatments according to ANOVA and LSD test, *n* = 3. LED lamps used: WL, white light; BL, blue light; GL, green light; RL, red light.*

### Photosynthetic Parameters

**FIGURE 4:**
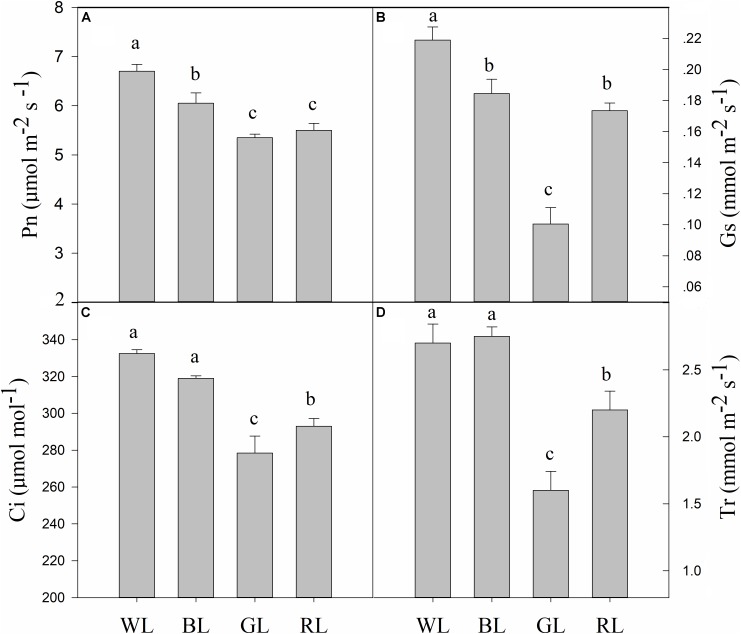
Effects of light quality on gas exchange [Pn **(A)**, Gs **(B)**, Ci **(C)**, and Tr **(D)**] in *C. paliurus* (Means ± SD). Different letters indicate significant differences (*P* < 0.05) between LED light treatments according to ANOVA and LSD test, *n* = 3.

BL, GL, and RL treatments reduced *C. paliurus* plant photosynthesis compared to WL as indicated by the lower values of Pn, Gs, Ci, and Tr (Figure 4). The lowest values of Pn, Gs, Ci and Tr were obtained from GL-treated plants. Meanwhile, there were no significant differences in Ci and Tr between BL and WL.

### Chlorophyll Contents

Plants grown under WL were characterized by the highest total pigment content, Chla content, and Chlb content (Table 4). The carotenoids (Car) contents under light treatments differed, and the lowest value was observed from GL. Meanwhile, the plants grown under BL differed from WL by reduced Chlb content but not reduced Chla or Car content. The Chla/Chlb ratio increased under GL, while the lowest ratio was obtained from WL (Table 4). The Car/Chl ratio was the lowest under WL and was slightly higher under BL, but the highest value was observed under GL.

**Table 4:** Chlorophylls levels, Chlorophyll a/b and Car/Chl in *C. paliurus* exposed to different light quality (Means ± SD).

Treatment	Total chlorophyll content (mg⋅g^-1^)	Chla content(mg⋅g^-1^)	Chlb content(mg⋅g^-1^)	Car content (mg⋅g^-1^)	Chlorophyll *a/b*	Car/Chl
WL	5.1 ± 0.06a	2.6 ± 0.01a	2.5 ± 0.06a	1.07 ± 0.005a	1.1 ± 0.03d	0.21 ± 0.002c
BL	4.0 ± 0.04b	2.6 ± 0.01a	1.4 ± 0.03b	1.10 ± 0.005a	1.8 ± 0.04c	0.28 ± 0.002b
GL	2.8 ± 0.02d	2.0 ± 0.01c	0.8 ± 0.01d	0.83 ± 0.013c	2.5 ± 0.01a	0.30 ± 0.006a
RL	3.5 ± 0.17c	2.4 ± 0.08b	1.1 ± 0.09c	1.02 ± 0.036b	2.1 ± 0.10b	0.29 ± 0.005a


*Different letters indicate significant differences (*P* < 0.05) between LED light treatments according to ANOVA and LSD test, *n* = 3. LED lamps used: WL, white light; BL, blue light; GL, green light; RL, red light.*

### Chlorophyll Fluorescence Parameters

**FIGURE 5:**
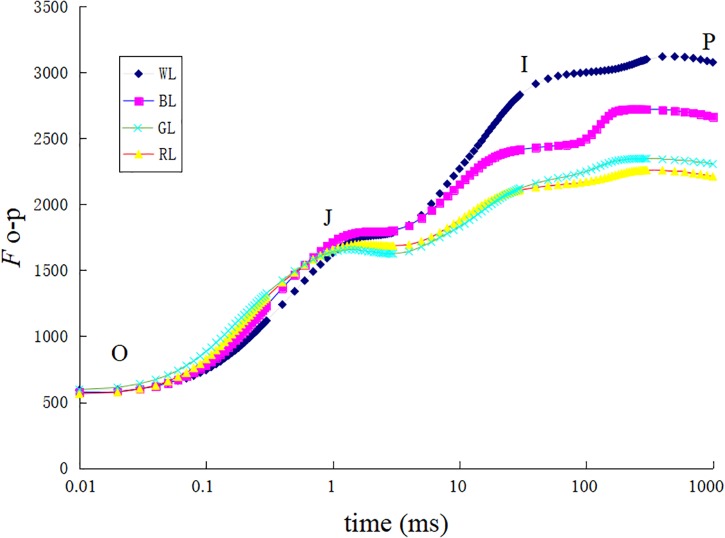
Chlorophyll a fluorescence transient of dark adapted leaves exposed to different LED light treatments.

**FIGURE 6:**
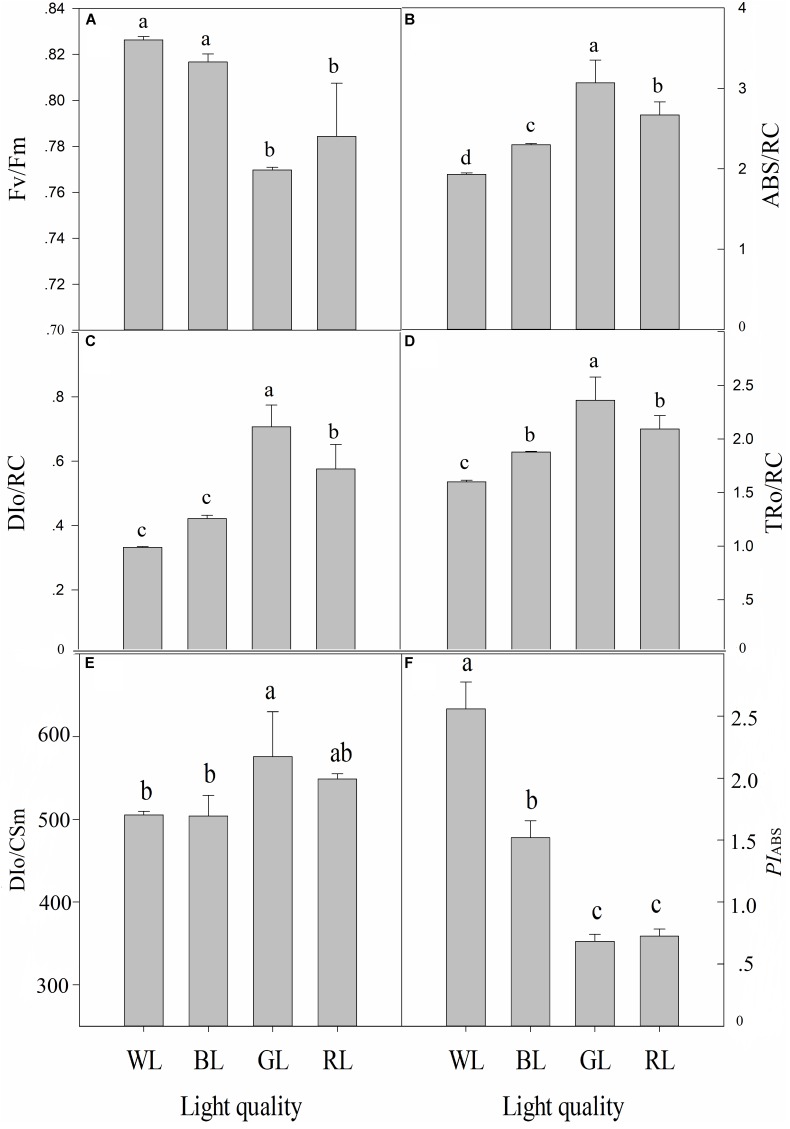
Effects of light quality on chlorophyll fluorescence parameters [Fv/Fm **(A)**, ABS/RC **(B)**, DIo/RC **(C)**, TRo/RC **(D)**, DIo/CSm **(E)** and *PI*_ABS_ **(F)**] in *C. paliurus* leaves (Means ± SD). Different letters indicate significant differences (*P* < 0.05) between LED light treatments according to ANOVA and LSD test, *n* = 3.

The increase in leaf fluorescence transients in *C. paliurus* plants under different light treatments showed a typical OJIP shape (Figure 5). Under GL and RL treatments, it showed repressed fluorescence transients, particularly at step I and P. The maximum quantum yield of PSII (Fv/Fm) was highest under WL (0.83 ± 0.002), slightly lower at BL (0.82 ± 0.004), lower under RL (0.78 ± 0.023), and lowest under GL (0.77 ± 0.001) (Figure 6). Besides, there was no significant difference in this parameter between WL and BL treatments. The light energy absorbed by one active reaction center (ABS/RC) was maximal under GL (3.1 compared with 2.3 in BL leaves, 2.7 in RL leaves and 1.9 in WL leaves) (Figure 6). In addition, non-photochemical quenching per one reaction center of PSII (DIo/RC) or per cross section (DIo/CSm) was the highest in the GL-treated leaves, which means that the energy dissipated were the highest in plants grown under GL (Figure 6). The trapped energy used to reduce primary quinone acceptor (Q_A_) by the unit reaction center of PSII (TRo/RC) was highest under GL and lowest under WL (Figure 6). Compared with WL and BL treatments, significantly lower values of photosynthetic performance index (*PI*_ABS_) were observed under GL and RL treatments (Figure 6).

### Lipid Peroxidation and H_2_O_2_ Content

**FIGURE 7:**
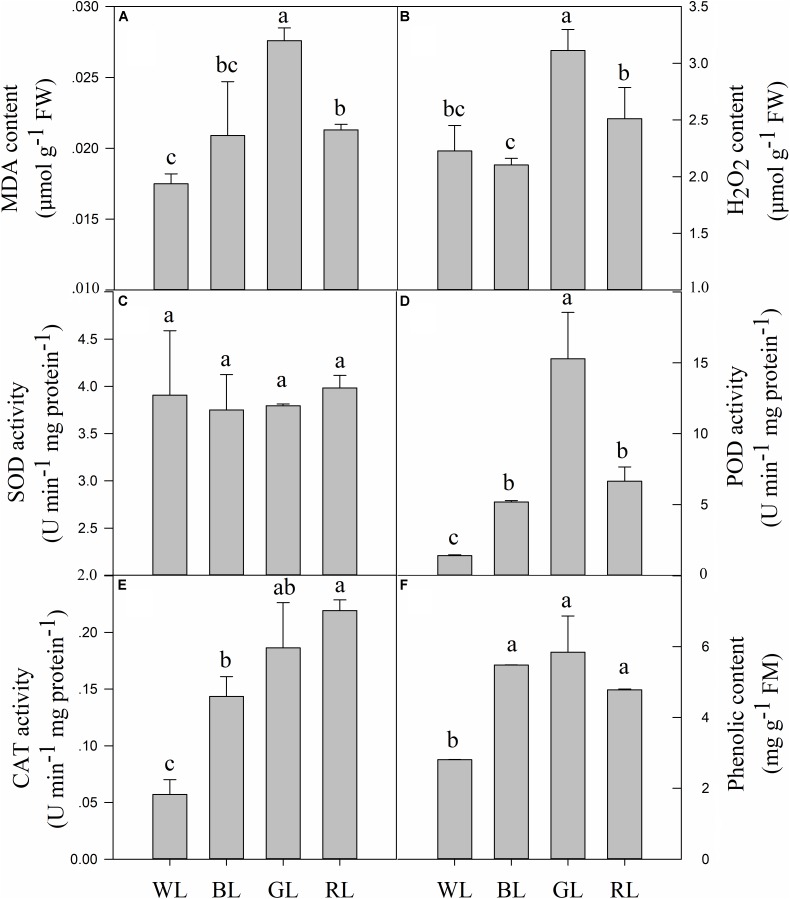
Effects of light quality on MDA content **(A)**, H_2_O_2_ content **(B)**, SOD activity **(C)**, POD activity **(D)**, CAT activity **(E)** and phenolic content **(F)** in *C. paliurus* leaves (Means ± SD). Different letters indicate significant differences (*P* < 0.05) between LED light treatments according to ANOVA and LSD test, *n* = 3.

The levels of MDA and H_2_O_2_ largely depended on the LED light used (Figures 7A,B). GL and RL caused severe lipid peroxidation of *C. paliurus* plants, compared to WL. MDA content and H_2_O_2_ were increased by 57.7 and 39.8% (*P* < 0.05) under GL, by 21.7 and 12.8% (*P* < 0.05) under RL, compared to WL (Figures 7A,B). No significant differences in MDA and H_2_O_2_ contents were observed between BL and WL treatments.

### Antioxidant Capacities

Changes in light quality did not have a significant effect on the SOD enzyme activity (Figure 7C). However, activities of POD and CAT were significantly affected by different light qualities (*P* < 0.05) (Figures 7D,E). POD and CAT activities increased by 271.2 and 151.3% (*P* < 0.05) under BL, by 994.2 and 226.4% (*P* < 0.05) under GL, and by 376.0 and 283.9% (*P* < 0.05) under RL, compared to WL.

### Responses of Phenolic Compounds

Contents of total phenolic compounds (including all individual phenolics detected by the HPLC system) in *C. paliurus* leaves showed a considerable variation in response to the changing LED light conditions (Figure 7F). BL, GL and RL caused a significant increase in the content of total phenolic compounds, compared to WL (*P* < 0.05). Besides, there were no significant differences in this parameter between BL, GL, and RL light conditions.

## Discussion

Light quality is a critical factor regulating photosynthetic capacity which directly affects the final yield and quality of plants. However, the responses to differential light quality are often species specific (Haliapas et al., 2008; Fan et al., 2013; Yu et al., 2017). Plants have evolved a system of sensory photoreceptors to accept changes of light signals in the environment (Gyula et al., 2003). The perception and regulation of these signals are controlled by a network of photoreceptors, such as cryptochromes (blue/UV-A light receptors, 340–520 nm), phytochromes (red/far-red receptors, 680–735 nm), and phototropins (phot1 and phot2) (Lin, 2000). Thus, when plants are grown under light with single wavelength band, some of the photoreceptors responsible for plant growth and morphogenesis will not receive signal, and this could lead to violations in plant development. In the present study, GL and RL significantly inhibited plant growth of *C. paliurus*, whereas positive effects were observed from WL and BL. Results of this study support our hypotheses that the difference in photosynthetic efficiency of *C. paliurus* plants under differential light quality is related to the degree of photoinhibition and the activation of photoprotection, which occur at both morphology and photochemical levels. And these photoprotective mechanisms are mainly related to better morphogenetic occurrences like leaf growth and stomata development, effective regulation of photosynthetic pigments and photochemistry activity, as well as reduced ROS accumulation.

Light quality affects a range of plant characteristics, such as seedling height, biomass accumulation, leaf size and anatomical structure (Yu et al., 2017). Leaf movement, such as leaf rolling, is an efficient strategy to protect leaves from photodamage (Corlett et al., 1994). However, leaf movement wasn’t observed in *C. paliurus* plants under differential light quality in this study. Compared with GL and RL, *C. paliurus* plants under WL and BL performed much better with higher values of total biomass, SLM, seeding height increment (SHI), leaf thickness and palisade length (Tables 1, 2 and Figure 2). Considering the white LED used containing blue light (445nm), we can conclude that blue light is more important for *C. paliurus* growth at seedling developmental stage than other lights. This result was inconsistent with those reported in other plants like strawberry, *Cistanche deserticola* and *Anoectochilus roxburghii* (Ouyang et al., 2003; Miranda and Williams, 2007; Ye et al., 2017). Some previous studies have revealed that a higher biomass allocation to the root system and a higher value of root/shoot ratio can increase the ability of plants to survive under stress conditions (Aranda et al., 2001; Tsakaldimi et al., 2005; Luis et al., 2010). Thus, the significantly higher values of root/shoot ratio under BL, GL, and RL than that under WL indicated that these lights caused varying degrees of stress on *C. paliurus* seedlings, among which the highest was observed under GL (Table 1). Compared with WL and BL, RL used in this study had a significantly inhibitory effect on plant height, LA, and biomass of *C. paliurus* seedlings, which was inconsistent with that of cucumber (Su et al., 2014). However, RL treatment caused a significant increase in GDI of *C. paliurus* than other lights used (Table 2). This result was different from what is reported on *Sinapis alba, Pelargonium* and *Camptotheca acuminata*, of which RL resulted in a higher allocation to plant shoot growth (Cosgrove, 1982; Appelgren, 1991; Yu et al., 2017). These contrast findings confirm that the responses of plant morphological characteristics to changes of light quality are species specific.

Stomata regulate water vapor diffusion and carbon dioxide uptake in plants. Stomatal density, distribution and opening thus affect plant photosynthesis, which are often influenced by many factors including light, temperature, and CO_2_ (Ye et al., 2017). Blue light has been reported to cause the voltage-dependent plasma membrane K^+^ channels to open, which then enhances water and K^+^ flow into the guard cells, and finally forces the stomata to open (Schroeder et al., 2001; Shimazaki et al., 2007). In the current study, values of leaf stomatal length, width and opening were observed largest under BL (Table 3 and Figure 3), which were similar to those reported in grape, *Tagetes erecta*, and *Anoectochilus roxburghii* (Heo et al., 2002; Poudel et al., 2008; Ye et al., 2017). The decreases in Pn associated with the reduced Tr, Gs, and Ci under RL and GL indicated that stomatal limitation occurred (Figure 4), which was inconsistent with the lower values observed in stomatal length, width, single stomatal pore area, and number of stoma per unit LA (Table 3 and Figure 3). The lower stomatal opening and number per unit LA under RL and GL might help plants to reduce the burden of the PSII reaction center and protect tissues from light stress (Lawson et al., 2011). The percentage absorption of GL (70–80%) by plant leaves has been reported not much smaller than that of BL and RL (90%), GL is absorbed very weakly by pigments and chlorophylls but it can reach the deeper layers of the leaf (Terashima et al., 2009). Thus, it is possible that the excess energy (when PPFD kept the same at 850 μmol m^-2^ s^-1^) that can’t be utilized by photochemistry then causes photoinhibition. A decrease in growth with an increase in the proportion of GL has also been demonstrated by Kim et al. (2004a,b). The highest ratio of palisade to spongy in GL treatment (Table 2 and Figure 2) also suggested that plants under green light changed their leaf anatomy to adapt to the light stress especially when stomatal closure happened and Ci became limited (Figures 3, 4). However, we also noticed that although plants under RL showed much higher Gs than under GL, the net rate of CO_2_ assimilation did not differ between them (Figure 4). As a result, the differences in Pn among all types of plants cannot be explained by the change in Gs, and non-stomatal limitations might exist. Thus, the quantitative analysis of photosynthetic limitations under different light qualities should be carried out in the future.

Chlorophyll content is another important factor for photosynthesis and plant growth. In this study, differential light quality significantly affected chlorophyll pigments and their ratios (Table 4). The decreases in total chlorophyll content were observed under BL, GL and RL, suggesting that these lights caused damages to photosynthetic pigments. Yu et al. (2017) showed that RL strongly promoted Chlb synthesis in *Camptotheca acuminata*. Similar results were also reported in *Cattleya loddigesii* and *Triticum aestivum* (Galdiano et al., 2012; Manivannan et al., 2015). However, compared with RL, BL treatment in this study resulted in a significantly higher leaf chlorophyll content, which was inconsistent with that reported in lettuce, *Toona sinensis* and *Anoectochilus roxburghii* (Zhang et al., 2010; Kobayashi et al., 2013; Ye et al., 2017). Chla is mainly concentrated around PSI and PSII, whereas Chlb is most abundant in light-harvesting complexes (LHC) (Venema et al., 2000). Thus, a change in the Chla/b ratio often indicates a change in the ratio between the light-harvesting complexes (LHC) and the reaction centre complexes of the photosystem. The significantly increased values of Chla/b ratio under BL, GL and RL showed that plants under these lights changed the ratio of photosynthetic pigments to maintain a good photoelectron transfer system.

Carotenoids play an essential photoprotective role in chloroplasts by scavenging ROSs (Schmitt et al., 2014). Furthermore, the photoprotective role of xanthophylls for dissipation of excess energy has been demonstrated (Tefler, 2002). Thus, the significantly higher carotenoids contents of *C. paliurus* plants under WL and BL can lead to the lower levels of ROSs compared to other light quality, which might help plants to maintain a better growth rate. Car/Chl is another important indicator for the adaptation of plants to light conditions, as it is known that increases in this parameter are often observed when plants are grown under stress (Penuelas et al., 1995). Thus, compared with WL and BL, GL and RL have caused the unhealthy development of *C. paliurus* plants. Since Car values between WL and BL did not differ significantly and were significantly lower under RL and GL, we could conclude that the decreases in total chlorophyll contents contributed the most to the significantly higher Car/Chl ratios under BL, GL and RL.

Chlorophyll fluorescence serves as a subtle reflection of the photochemical activities of photosynthetic systems in plant physiological experiments (Pashkovskiy et al., 2018). Generally, plants grown under light stress typically have lower Fv/Fm values than non-stressed ones (Baker, 2008). In the present study, Fv/Fm, *PI*_ABS_ and Pn were significantly reduced under RL and GL compared with WL and BL (Figures 4, 6), indicating the occurrence of significant photoinhibition under these light wavelengths. The light energy absorbed by one active reaction center (ABS/RC) usually reflects the ratio between chlorophyll pigments in the fluorescence-emitting complexes and in the active reaction centers (Strasser et al., 2010). The increase in ABS/RC in plants under GL and RL can be explained by a decrease in the number of active reactive centers of PSII, which might serve as a defense mechanism to reduce the burden of its systems when light stress occurred. Another effective protective mechanism is the dissipation of the absorbed light energy into heat (NPQ mechanism) (Baena-Gonzalez and Aro, 2002), which is confirmed by the much higher values of non-photochemical quenching per one reaction center of PSII (DIo/RC) or per cross section (DIo/CSm) in *C. paliurus* plants under GL and RL (Figure 6). Furthermore, the energy used to reduce Q_A_ by the unit reaction center of PSII (TRo/RC) was observed significantly higher under GL and RL, which indicated that *C. paliurus* plants under these lights improved their efficiency of the remaining active reaction centers to dissipate the energy in the electricity chain. It seems that plants grown under GL in this study have developed most of the characteristics of so called “shade plants” – they have thinner leaves, larger LA, thin palisade tissue, lower number of stomata, and higher chlorophyll a/b ratio. These changes in morphological, chlorophyll content and proportion provide an effective photoprotective strategy for plant growth, as mentioned above. However, the question remains if the observed decrease of PSII capability is reversible, i.e., it is photoinactivation, or irreversible - photoinhibition, but this needs to be further studied.

ROS, such as O_2_•^-^, H_2_O_2_ and •OH, can be generated by the direct transfer of the excitation energy from chlorophyll, electron transport chains and the chloroplasts (Sriram et al., 2005), which can cause photodamage to PSII directly (Pashkovskiy et al., 2018). Higher values of MDA (a factor which reflects the level of lipid peroxidation) and significantly higher values of H_2_O_2_ were observed from plants under GL and RL (Figure 7), which indicated that a high level of oxidative damage had been caused by excessive ROS. It is interesting to find that *C. paliurus* plants under BL also accumulated more phenolics to help maintain the ROS balance (Figure 7F). The neighboring hydroxyl groups of phenolics allow them to act as an effective role to reduce ROS (Cruces et al., 2017; Liu et al., 2018c). ROS scavenging abilities in plants under GL and RL were confirmed mainly through other methods such as increasing enzymatic antioxidants [peroxidase (POD), and catalase (CAT)] (Figures 7D,E), and also phenolics (Figure 7F). These results indicated that increasing enzymatic antioxidants and phenolics was an effective measure to scavenge ROS especially when light stress occurred.

## Conclusion

Growth and photosynthesis of *C. paliurus* plants showed highest performances under WL and BL, which were attributable to the better morphogenetic occurrences like leaf growth and stomata development, regulations of photosynthetic pigments and photochemistry activity, as well as reduced ROS accumulation. Plants subjected to GL and RL suffered significant photoinhibition but also effectively developed photoprotective mechanisms mainly through changes of leaf anatomy and pigment ratios, NPQ mechanism and increasing enzymatic antioxidants and phenolics contents. This study will help uncover the response mechanisms of plant photosynthesis to light quality, and provide a theoretical basis for improving the cultivation of *C. paliurus* seedlings. The underlying molecular mechanism remains unknown and more tests in both greenhouse and field conditions, such as adding suitable plastic films or LEDs with spectral characteristics at seedling developmental stage, are needed for applications to improve the yield and quality of this species.

## Author Contributions

YL is responsible for the whole process of experimenting and writing the paper. TW and SF provide experimental guidance and amend the manuscript. MZ and JQ help to do the experiment.

## Conflict of Interest Statement

The authors declare that the research was conducted in the absence of any commercial or financial relationships that could be construed as a potential conflict of interest.
