# Supplementary material for: Responses of Morphology, Gas Exchange, Photochemical Activity of Photosystem II, and Antioxidant Balance in Cyclocarya paliurus to Light Spectra
Source: Front Plant Sci. 2018 Nov 21;9:1704. doi: 10.3389/fpls.2018.01704 (PMC6258815; doi:10.3389/fpls.2018.01704)
Supplement: Supplementary file 1 [file Data_Sheet_1.docx]

Supplementary Material

**Responses of morphology, gas exchange, photochemical activity of photosystem II, and antioxidant balance in *Cyclocarya paliurus* to light spectra**

**Yang Liu^1^,Tongli Wang^2^****,Shengzuo Fang^1,3*^,Mingming Zhou^1^,Jian Qin^1^**

*** Correspondence:** Shengzuo Fang: fangsz@njfu.edu.cn

## Supplementary Figures

**
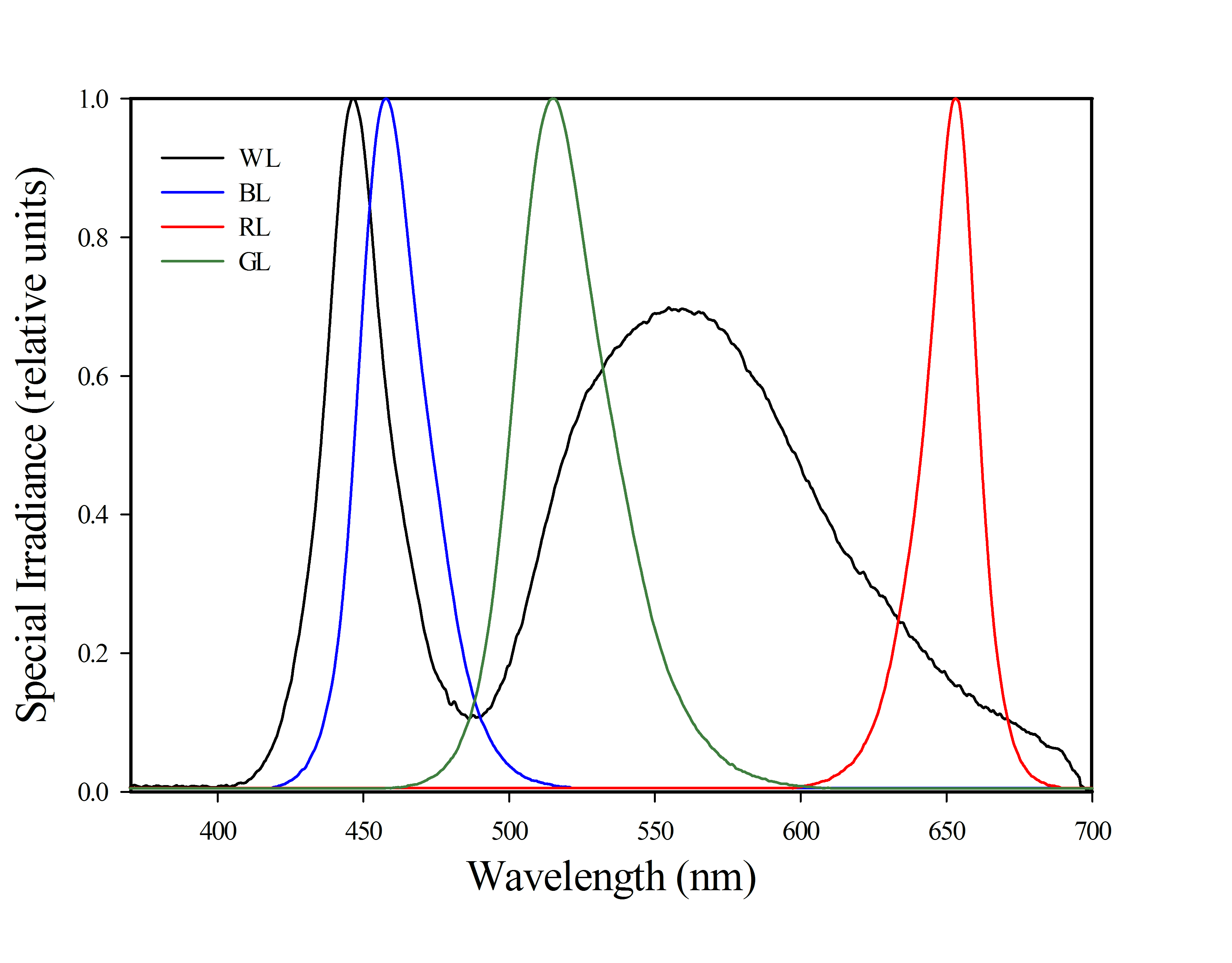
**

**Supplementary Figure 1.** Relative spectral irradiance of the LED lights used: WL=white light, BL=blue light, GL=green light, RL=red light.


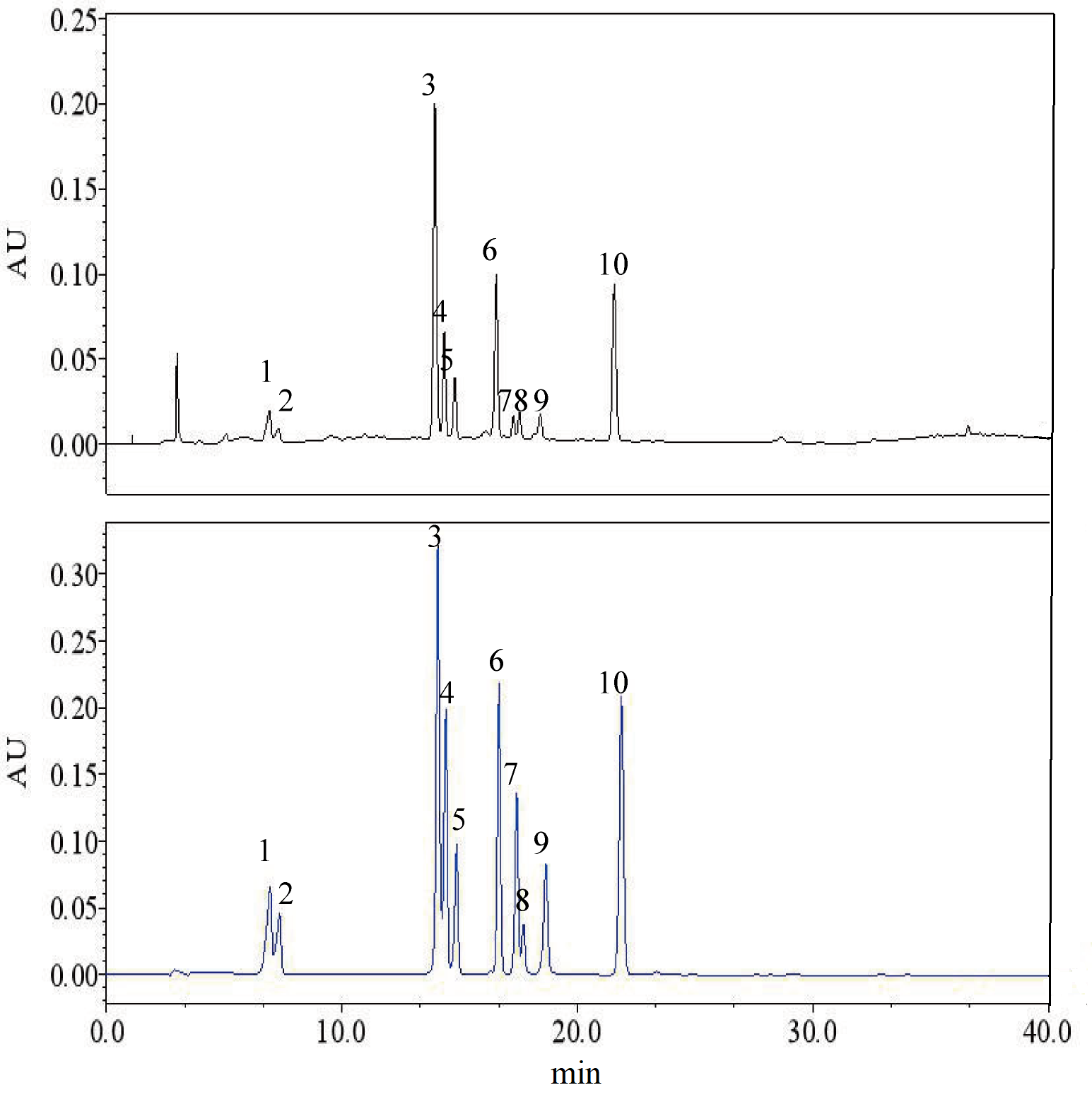


**Supplementary Figure 2.** HPLC chromatograms of a representative sample (top) and a mixed standards (bottom) for phenolics. 1. 3-*O*-caffeoylquinic acid; 2. 4-*O*-caffeoylquinic acid; 3. quercetin-3-*O*- glucuronide; 4. quercetin-3-*O-*galactoside; 5. isoquercitrin; 6. kaempferol-3- *O*-glucuronide; 7. kaempferol-3-*O*-glucoside; 8. quercetin-3-*O*-rhamnoside; 9. 4,5-di-*O*-caffeoylquinic acid; 10. kaempferol-3-*O*-rhamnoside.
